# A combined healthy lifestyle score in relation to glioma: a case-control study

**DOI:** 10.1186/s12937-022-00758-0

**Published:** 2022-01-19

**Authors:** Soraiya Ebrahimpour-Koujan, Mehdi Shayanfar, Minoo Mohammad-Shirazi, Giuve Sharifi, Ahmad Esmaillzadeh

**Affiliations:** 1 Department of Community Nutrition, School of Nutritional Sciences and Dietetics, Tehran University of Medical Sciences, Tehran, Iran; 2 Department of Clinical Nutrition, School of Nutritional Sciences and Dietetics, Tehran University of Medical Sciences, Tehran, Iran; 3 Department of Clinical Nutrition and Dietetics, National Nutrition and Food Technology Research Institute, Shahid Beheshti University of Medical Sciences, Tehran, Iran; 4 Department of Neurosurgery, Loghman Hakim Hospital, Tehran, Iran; 5 Obesity and Eating Habits Research Center, Endocrinology and Metabolism Molecular -Cellular Sciences Institute, Tehran University of Medical Sciences, Tehran, Iran; 6 Department of Community Nutrition, School of Nutrition and Food Science, Isfahan University of Medical Sciences, Isfahan, Iran

**Keywords:** Healthy life style score, Glioma, FFQ, Case-control

## Abstract

**Background:**

The evidence on the association between adherence to a healthy lifestyle and risk of glioma are scarce. This is particularly relevant to Middle Eastern countries where lifestyle factors including dietary intakes, physical activity and environmental contributors are different from other parts of the world. The aim of this case-control study was, therefore, investigating the association between adherence to a healthy lifestyle and odds of glioma among adults.

**Methods:**

Totally, 128 newly diagnosed glioma cases and 256 age- and sex-matched controls were recruited in this hospital-based case-control study. Dietary intakes were examined by the use of a 126-item validated FFQ. International Physical Activity Questionnaire (IPAQ) was used for measuring physical activity of participants. To construct a healthy lifestyle score (HLS), data from dietary intakes, physical activity and BMI were used. Subjects in the low risk categories of the mentioned components received the score of 1, otherwise they received the score of 0. The final HLS was computed through summing up the scores of components.

**Results:**

After adjustment for age and sex, we found that individuals with the highest HLS score were 55% less likely to have glioma compared with those with the lowest score (OR: 0.45; 95% CI: 0.22, 0.92). Additional controlling for other potential confounders made the association stronger (OR: 0.28; 95%CI: 0.12, 0.66). In terms of individual components of healthy lifestyle score, subjects with a healthy diet had 54% lower odds of glioma than those with a non-healthy diet (OR: 0.46; 95%CI: 0.26, 0.80). No significant associations were seen between physical activity level or BMI status and glioma.

**Conclusion:**

We found evidence indicating that adherence to a healthy lifestyle, in particular a healthy diet, was associated with a lower odds of glioma. Prospective cohort studies are needed to confirm these findings.

## Introduction

Glioma is a heterogeneous primary brain tumor with multiple subtypes. Its histologic range is from Glioblastoma multiform, a most common aggressive tumor, to low grade gliomas [1]. Gliomas account for almost 27% of brain tumors and the overall incidence of malignant glioma is 7.2 per 100,000 in general population [1]. According to the report of National Cancer Registry (NCR) in Iran, the overall incidence rate for malignant brain tumors is 2.74 per 100,000 in general population and high grade gliomas are 60.4% of the primary malignant registered brain tumors [2, 3].

Due to poor prognosis of glioma, prevention is of high priority. Several contributors to glioma are still unknown. In addition to genetic, lifestyle-related variables have been individually linked with the risk [4, 5]. For instance, several nutrients and foods and even dietary patterns have been associated with the risk [6]. It had been suggested that a healthy diet rich in antioxidants and phytochemicals may likewise mitigate glioma malignancy through their anti-inflammatory properties [7–9]. The modern researches represented the increasing efficacy of micronutrients through their synergistic interactions. Such interaction of nutrients displays pleiotropic effects against glioma [10, 11]. They simultaneously affect different mechanisms involved in malignancy initial results indicating enhanced anticancer efficacy. This anti-carcinogenic group of nutrients prevents carcinogens from damaging cellular macromolecules such as DNA [10]. Physical activity levels have also been associated with the risk of glioma in some studies. In addition, smoking and smoke exposure was associated with increasing risk of glioma [8, 9, 12]. Findings from some prospective cohort studies have also introduced overweight and obesity as a contributing risk factor to glioma [13, 14]. Although, retrospective studies have shown associations of glioma with many different type of nutrients and food groups, findings are generally inconclusive. Despite huge evidence of the link between individual lifestyle related variables including natural compounds found in herbs, fruits, vegetables etc. and risk of glioma [15, 16], we are aware of no study that considered the combined lifestyle factors in relation to glioma. Moreover, some primary studies have shown that smoking can contributes to the chance of glioma, but such association was not found in a large meta-analysis [17]. Due to complex etiology, the association of nutritional and lifestyle factors with the incidence of gliomas are still not clearly identified and described. Examining the association of whole lifestyle factors with glioma can help identifying the percentage of this condition that might be prevented through improving lifestyle. Combined lifestyle factors including dietary intakes, BMI, physical activity, etc. have earlier been examined in relation to mortality and several cancers including breast, prostate and pancreatic cancer [18–21]. Adherence to healthy lifestyle was inversely associated with cardiovascular mortality, lethal prostate, pancreatic and breast cancer [19–22].

Lifestyle factors are different in Middle Eastern countries than other parts of the world. There are alarming levels of obesity prevalence, lack of physical activity, low consumption of whole grain, fruit and vegetables among Middle Eastern people [23–25]. Unlike those in western countries, people in the Middle East take high percentage (more than 60%) of their energy from carbohydrates and intakes of refined grains are in highest amounts in these regions compared with other part of the world [23, 24, 26]. Trans fats are responsible for almost 4% of energy intake in Middle Eastern countries, while consumption of proteins, fruit and vegetables is not so much. These nutrients and foods are the components of Healthy Eating Index (HEI) which we used it to develop the Healthy Life Style score [23–25]. Smoking among women in these countries is not comparable to that in western nations. Adult female smoking prevalence varies greatly: in more developed, westernized countries the mean female smoking prevalence is 17.2%, whereas in developing nations it is 3.1% [27–29]. Middle Eastern adults tolerate a high level of stress and psychological distress (5.6% of total disease) due to several social, economic and political situations [30]. It has been shown that the prevalence of psychological disorders in this region is related to obesity, diabetes and other chronic disease [31, 32]. Cultural expectations in these countries resulted in low levels of physical activity, in particular among women [33]. Some women in these countries think that physical activity is the masculine activity or think it is a waste of time. Some women shame to do exercise. There is no family education to do exercise from childhood among women and there is no enough education in the society. The lack of physical activity puts these people at the risk of obesity and chronic disease [34, 35]. According to our literature review, healthy life style score consists of dietary components, physical activity, BMI, smoking and psychological disorders [18–21]. However, this score was different between studies regarding all of these components, some of them had considered there or more factors. Taken together, these points have been resulted in a different lifestyle in these countries compared to developed nations. Therefore, assessment of combined lifestyle factors in this region in relation to cancer as one of chronic conditions might provide some new information and additional reasons for the different prevalence of chronic diseases in this region compared with those in other parts of the world. This case-control study was, therefore, conducted to investigate the association between adherence to healthy lifestyle and risk of glioma among Iranian adults.

## Participants and methods

### Participants

This project was a hospital-based case-control study carried out on newly diagnosed patients (maximum one month elapsed since the detection) and healthy controls in Tehran, Iran, between 2009 and 2011. We selected participants, both cases and controls, using convenience-sampling method from the hospitals affiliated to Shahid Beheshti University of Medical Sciences. As the whole project was designed to assess the association of major dietary patterns with glioma, the sample size was calculated considering the amount of fruit and vegetables consumption in Iranian population. Based on the results of a national study, approximately 60% of adult population in the country had lower than the recommended levels of fruits and vegetables [26]. We hypothesized that low fruit and vegetable intakes would double the risk of glioma [9]. Considering 80% study power along with type I error of 0.05 and the desired CI of 95%, the minimum required sample size was calculated to be 115 cases and 230 healthy control subjects. To reduce errors, we enrolled 128 cases and 256 controls aged 20–75 years old using convenience-sampling method. Based on other lifestyle factors, it seems that this sample size is sufficient for the current study. Similar to present project, other publications have been derived from this dataset. Considering the findings of previous studies, this sample size showed a good expected association in previous publications [36–38]. Therefore, it seems that this sample size is sufficient for the life style factors. Cases were individuals with pathologically confirmed glioma (ICD-O-3 morphology codes 9380–9481) during the previous month that had been referred to Neurosurgery department of the hospitals. Controls were healthy individuals who were recruited from orthopedic and surgical departments of the same hospitals or were outpatients referring to the same clinics. The participation rate was 100% among cases and 89% among controls. Cases and controls were individually matched by age (±5) and sex. Individuals with a history of any type of pathologically confirmed cancer (except glioma), chemotherapy and radiotherapy (due to cancer) were not included in this study. All cases and controls provided written informed consent. The study was ethically approved by the Medical Ethics Committee of the Tehran University of Medical Sciences, Tehran, Iran.

### Dietary assessment

Dietary intakes of participants during a year before the diagnosis of glioma in cases and during a year before the interview in controls were evaluated by a validated Block-format 123-item semi-quantitative FFQ. The FFQ was consisted of 123 food items with standard portion sizes commonly consumed by Iranian people. FFQ was completed for both cases and controls by the cooperation of individuals (the patients’ parents or wives or relatives or care-takers) who were involved in the preparation and cooking of foods. All questionnaires were completed by trained interviewers through face to face interviews. The interviewers were the same for cases and controls. Reported consumption frequencies were converted to grams per day by using household measures. Daily intakes of energy and nutrients were computed for each person by using the modified US Department of Agriculture food consumption database.

Findings from the validation study of this FFQ on 131 apparently healthy people aged 35–65 y revealed good correlations between dietary intakes assessed by FFQ and those obtained from 12 dietary recalls (one 24-h recalls per month) [39]. The reliability of FFQ was assessed by comparing nutrient intakes obtained from the FFQ on four occasions 3-month apart. The intra-class correlation coefficients for the reproducibility of the FFQ were 0.75 for carbohydrates, 0.76 for proteins and 0.72 for fat intakes. The correlation coefficients for the validity of FFQ, compared to the average of twelve 24-h dietary recalls, for dietary carbohydrate, protein and fat were 0.75, 0.76 and 0.65, respectively [39]. These data indicated that the FFQ provides valid measurements of long-term nutrient intakes [39].

### Construction of healthy lifestyle score (HLS)

To construct a healthy lifestyle score, data from dietary intakes, physical activity and BMI were used. With regards to a healthy diet, we used the Alternative Healthy Eating Index-2010 (AHEI-2010) [40]. The index was composed of 9 components [fruit, vegetables, whole grains, nuts and legumes, long chainomega-3 fats (docosahexaenoic acid and eicosapentaenoic acid), polyunsaturated fatty acids, sugar sweetened drinks and fruit juice, red and processed meats, and sodium] [40]. In the current study, alcohol and trans fatty acid consumption was not included into the score, due to lack of information in the original dataset. To construct the index, first we obtained energy-adjusted intakes of the mentioned components based on residual method [41]. Then, participants were classified based on the decile categories of energy-adjusted intakes of these components. Individuals in the highest deciles of fruits, vegetables, whole grains, nuts and legumes, long chain omega-3 fats and polyunsaturated fatty acids were given the score of 10 and those in the lowest decile of these items were given the score of 1. Individuals in the other deciles of these components were assigned the corresponding scores. Regarding sugar sweetened drinks and fruit juice, red and processed meat and sodium intake, the lowest decile was given a score of 10 and the highest decile was given the score of 1. Those in deciles 9, 8, 7, 6, 5, 4, 3 and 2 of these components were given the scores of 2, 3, 4, 5, 6, 7, 8 and 9, respectively. To compute the AHEI-2010, the scores of individual items were summed up. The final AHEI score varied from a minimum score of 9 to a maximum score of 81. Participants in the highest 40% of AHEI-2010 (upper two fifths) were considered as having a healthy diet.

In terms of physical activity, International Physical Activity Questionnaire (IPAQ) was used for measuring physical activity of participants through the face to face interviews. All results of the IPAQ were expressed as Metabolic Equivalents-hours per week (MET-h**/**week). Participants were then classified into tertiles in terms of their MET-h**/**week values. We defined low risk group as individuals with active (highest tertile) and moderately active (middle tertile) lifestyle.

**Fig. 1:**
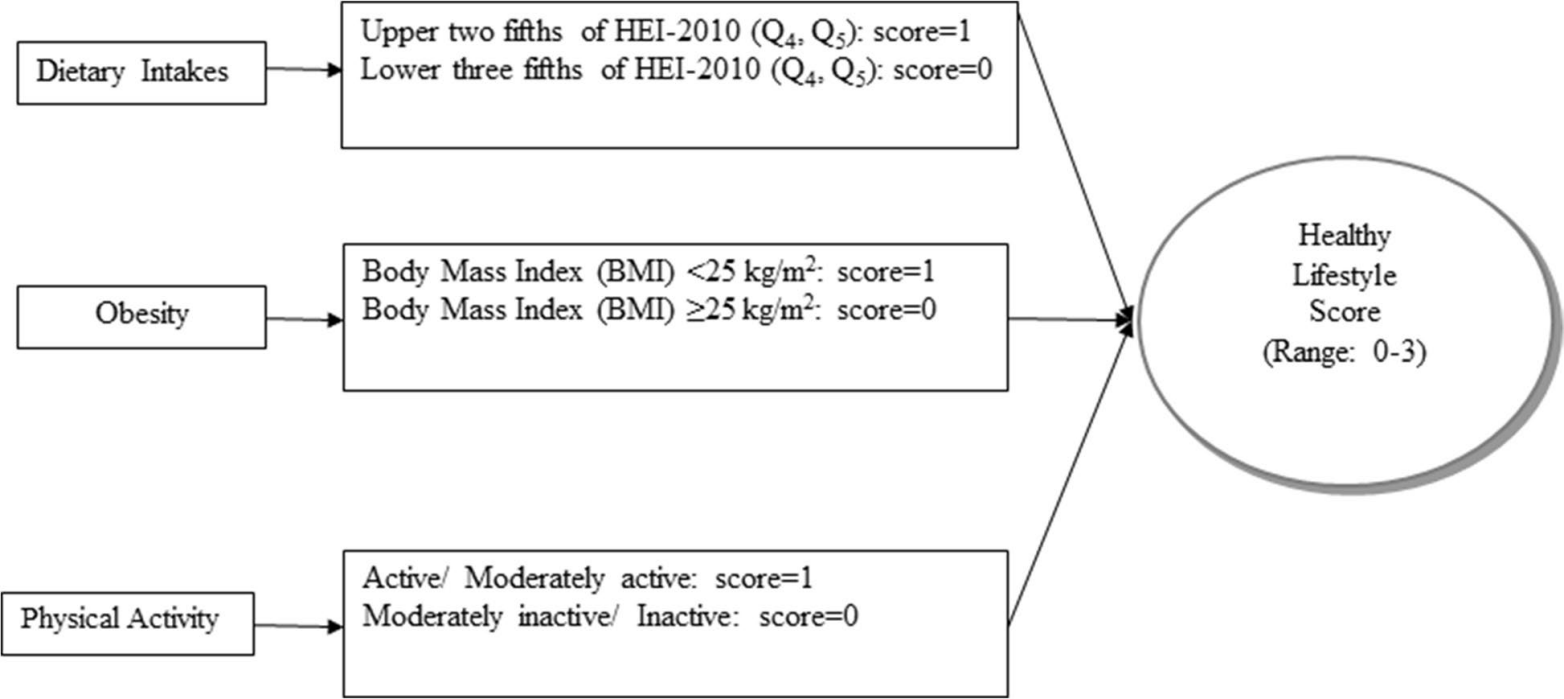
The healthy lifestyle development

Body weight was quantified by a digital scale to the nearest 500 g with the subjects wearing the light clothing and no shoes. Height was measured by a tape measure to the nearest 0.5 cm in standing status while the subjects’ shoulders were in a normal position. Body Mass Index (BMI) was calculated as weight in kilogram divided by height in meters squared. Individuals with BMI < 25 kg/m^2^ were defined as healthy. The final HLS was constructed through summing up the scores that each participant obtained for components of lifestyle as mentioned above, given that subjects in the low risk categories of the mentioned components received the score of 1, otherwise they received the score of 0. Therefore, a composite global healthy lifestyle score ranged from 0 to 3. Figure 1**.** shows how the healthy lifestyle score was developed.

### Assessment of glioma

Glioma was diagnosed based on pathological test by using International Classification of Diseases for Oncology third edition and morphology codes of 9380–9481 [42]. Only patients with a maximally one month of the confirmation of glioma were included in the study.

### Assessment of other variables

A pretested questionnaire was used to collect data on age, sex, marital status, place of residence, education, occupation, smoking status, use of supplements (Iron, Calcium and multivitamins), family history of cancers and glioma, history of allergy and trauma, history of hypertension, exposure to chemicals in the past 10 years (Formaldehyde, plastics, benzene, mercury, arsenic and lead), cooking methods, drug use (any type of medications), personal hair dye use, duration of cell phone use (years) and history of exposure to the radiographic X-ray. All measurements were completed by a trained dietitian. On the basis of previous studies, we considered farmers as having a high risk occupation for glioma [43]. Individuals who lived in places near the electromagnetic fields and cell phone and broadcast antennas in the last 10 years were defined as living in high risk areas [44]. Individuals who consumed any kind of fried foods at least twice per week were considered frequent fried food users. This definition was also used for barbecue use, microwave use [16, 45, 46] as well as consumption of canned foods [45].

### Statistical analysis

Before starting the analysis, we found that number of glioma cases in the highest category of HLS (i.e. HLS = 3) was very low. To avoid having wide CIs in this category, we decided to merge the highest two categories of HLS [2 and 3] and considered them as one category. General characteristics and dietary intakes of study participants across categories of the HLS scores were examined using one-way ANOVA for continues variables and chi-square for categorical variables. The association of HLS with glioma was assessed by using conditional logistic regression in different models. Age (continues) and sexes (male/female) were adjusted for in the first model. Additional controlling for family history of cancers (yes/no), family history of glioma, marital status (yes/no), education (university graduated/non-university graduated), high-risk occupation (farmer/non-farmer), high-risk residential area (yes/no), duration of cell phone use (continues), supplement use (yes/no), history of exposure to the radiographic X-ray (yes/no), history of head trauma (yes/no), history of allergy (yes/no), history of hypertension (yes/no), smoking status (smoker/non-smoker), exposure to chemicals (yes/no), drug use (yes/no), personal hair dye (yes/no) was done in the second model. Cooking methods including frequent fried food intake (yes/no), frequent use of barbecue (yes/no), canned foods and microwave (yes/no) were taken into account in the third model. All confounders were chosen based on previous publications. The statistical analyses were carried out by using SPSS version 18. *P* values were considered significant at < 0.05.

## Results

Main characteristics of the study participants in both case and control groups as well as across categories of the healthy lifestyle score are presented in Table 1**.** Having high-risk occupations, living in high-risk residential areas, and being frequently exposed to radiographic X-ray and chemicals were highly prevalent among cases than controls. Glioma patients were more likely to have a history of head trauma and family history of glioma. In addition, they were more likely to be frequent fried food consumers than controls. In contrast, cell phone use, microwave use, personal hair dye use and smoking were less prevalent among cases than controls. Also, cases had lower mean of healthy life style score than controls. Moreover, mean of Mean age, BMI and physical activity were not significantly different between the two groups. No other significant difference was seen in the distribution of participants in terms of other categorical variables sex, marital status, education level, any history of allergy, hypertension and family cancer, frequent use of barbecue, canned foods, drug and supplement use.

**Table 1:** General characteristics of study participants*

	Groups				Healthy lifestyle score		
	Cases (n 128)	Controls (n 256)	P^*^	0 (*n* = 38)	1 (*n* = 161)	2 (*n* = 184)	P^*^
Age (year)	43.4 ± 14.6	42.7 ± 13.3	0.65	46.1 ± 14.6	42.5 ± 14.3	42.7 ± 13.0	0.33
BMI (kg/m^2^)	26.2 ± 4.2	26.1 ± 3.8	0.75	29.0 ± 2.9	27.1 ± 3.8	24.8 ± 3.8	< 0.001
Physical activity (MET-h/week)	34.7 ± 6.3	33.8 ± 5.5	0.12	28.27 ± 2.4	32.7 ± 5.3	36.6 ± 5.4	< 0.001
Duration of cell phone use (years)	2.8 ± 2.9	3.7 ± 2.6	0.003	4.1 ± 2.4	3.7 ± 2.8	3.1 ± 2.6	0.03
Females (%)	41	42	0.99	44.7	39.8	42.9	0.775
Married (%)	79	80	0.66	89.5	80.7	76.6	0.125
University graduated (%)	12	17	0.22	15.8	16.8	13.6	0.708
High risk job† (%)	10	3	0.003	2.6	4.3	6.5	0.499
High-risk residential area‡ (%)	30	21	0.05	31.6	24.2	22.8	0.519
History of exposure to radiographic X-ray (%)	16	7.4	0.01	5.3	11.2	10.3	0.553
History of head trauma (%)	44	29	0.004	26.3	33.5	35.9	0.521
History of allergy (%)	25	29	0.40	39.5	31.1	22.8	0.058
History of hypertension (%)	2	5	0.28	2.6	3.7	4.9	0.762
Smoker (%)	16	25	0.02	23.7	23.6	20.1	0.709
Frequent fried food intake§ (%)	91	78	0.001	89.5	78.3	84.2	0.164
Frequent use of barbecue|| (%)	16	21	0.21	5.3	14.3	14.1	0.305
Frequent microwave use|| (%)	8	19	0.002	21.1	18.6	10.9	0.075
Frequent canned foods intake|| (%)	6	7	0.52	7.9	6.8	4.3	0.512
Drug use (%)	8	5	0.36	7.9	6.8	4.9	0.657
Personal hair dye use (%)	22	41	< 0.001	42.1	36.0	32.1	0.448
Exposure to chemicals (%)	20	11	0.01	10.5	11.8	15.8	0.477
Family history of glioma (%)	19	5	< 0.001	21.1	8.1	9.8	0.057
Family history of cancer (%)	33	34	0.90	34.2	32.9	33.7	0.983
Supplement use (%)	8	16	0.36	7.9	17.4	10.3	0.092
Healthy life style score	1.27	1.43	0.02				

* All values are Means±SD unless indicates; MET, metabolic equivalents

* *P* values were obtained from independent Student’s t test, one-way ANOVA or χ2 test, where appropriate

† Farmers were considered as having a high-risk occupation

‡ Individuals who lived in places near electromagnetic fields and cell phone and broadcast antennas in the last 10 years were defined as living in high-risk areas

§ Individuals who consumed fried food at least twice per week were considered as frequent fried food users

|| Individuals who used barbecue, microwave and canned foods at least twice per week were considered as frequent users

When we examined across categories of the healthy lifestyle score, individuals in the highest category were less likely to be obese and cell phone user and more likely to be physically active compared with those in the lowest category. There were no other significant differences in other variables across categories of HLS score.

Table 2 presents the dietary intakes of study participants separately by cases and controls as well as across categories of HLS score. Compared with controls, glioma patients had higher intakes of carbohydrates, vitamin B_12_, selenium, red and processed meat, fish, refined grains, whole grains and lower intakes of total fats, saturated fats, polyunsaturated fats, vitamin D, vitamin E, vitamin B_6_, potassium, calcium, fruits, vegetables, legumes and nuts, dairy products and salt. Adherence to healthy lifestyle behavior was associated with greater intakes of polyunsaturated fats, fruits, vegetables, legumes and nuts, dairy products and whole grain; and lower intakes of selenium, red and processed meat, refined grain and sugar sweetened beverages and partially white meat and salt.

**Table 2:** Dietary intakes of study participants

	Groups				Healthy lifestyle score		
	Cases (n 128)	Controls (n 256)		0 (n = 38)	1 (n = 161)	2 (n = 184)	
	Mean ± SD	Mean ± SD	P*	Mean ± SD	Mean ± SD	Mean ± SD	P*
Total energy (kcal/d)	2580 ± 560	2561 ± 722	0·72	2584 ± 670	2557 ± 708	2576 ± 643	0.957
**Nutrients**							
Carbohydrates (g/d)	425 ± 101	412 ± 128	0.02	422 ± 122	409 ± 110	421 ± 128	0.622
Proteins (g/d)	98 ± 22	97 ± 30	0.76	98 ± 25	98 ± 32	96 ± 22	0.824
Fats (g/d)	62 ± 19	66 ± 22	0.003	63 ± 21	66 ± 24	64 ± 17	0.712
Saturated fats (g/d)	19 ± 7	21 ± 9	0.02	19 ± 7	21 ± 10	20 ± 7	0.350
Polyunsaturated fats (g/d)	13 ± 4	14 ± 4	0.001	13 ± 4	13 ± 4	14 ± 4	0.037
Cholesterol (mg/d)	251 ± 141	235 ± 121	0.24	233 ± 81	253 ± 157	231 ± 59	0.288
Total fiber (g/d)	23 ± 11	23 ± 14	0.83	25 ± 13	24 ± 17	21 ± 9	0.074
Vitamin A (μg/d)	1353 ± 604	1397 ± 650	0.45	1447 ± 742	1338 ± 710	1404 ± 532	0.517
Vitamin D (mg/d)	1.3 ± 1	1.7 ± 1.1	0.002	1.5 ± 1	1.6 ± 1	1.6 ± 1	0.911
Vitamin E (mg/d)	5 ± 2	6 ± 3	0.015	5 ± 3	5 ± 3	5 ± 3	0.522
Vitamin C (mg/d)	126 ± 98	143 ± 113	0.09	129 ± 38	137 ± 137	139 ± 58	0.870
Vitamin B_6_ (mg/d)	1.9 ± 0.54	2 ± 0.76	0.047	2.0 ± 0.94	2.0 ± 0.80	1.9 ± 0.54	0.697
Folate (mg/d)	349 ± 90	382 ± 302	0.230	362 ± 111	386 ± 374	359 ± 78	0.597
Vitamin B_12_ (g/d)	10 ± 16	6 ± 4	0.001	8 ± 6	8 ± 14	6 ± 5	0.064
Methionine (g/d)	1603 ± 431	1555 ± 569	0.24	1546 ± 351	1607 ± 648	1546 ± 428	0.550
Potassium (mg/d)	4074 ± 783	4364 ± 1423	0.006	4074 ± 1126	4246 ± 1449	4322 ± 1081	0.553
Calcium (mg/d)	1020 ± 263	1139 ± 358	< 0.001	1068 ± 346	1089 ± 398	1113 ± 264	0.698
Zinc (mg/d)	12.4 ± 3	12.1 ± 4	0.26	11.8 ± 3	12.3 ± 3	12.2 ± 3	0.733
Copper (mg/d)	2.3 ± 0.7	2.4 ± 0.8	0.32	2.3 ± 0.71	2.4 ± 0.89	2.3 ± 0.61	0.873
Selenium (mg/d)	0.07 ± 0.04	0.06 ± 0.04	0.02	0.08 ± 0.04	0.07 ± 0.04	0.06 ± 0.03	< 0.001
**Food groups**							
Fruits (g/d)	325 ± 100	361 ± 124	0.001	326 ± 88	333 ± 117	367 ± 121	0.012
Vegetables (g/d)	258 ± 83	274 ± 86	0.04	246 ± 56	253 ± 79	287 ± 92	< 0.001
White meat (g/d)	30 ± 13	33 ± 22	0.16	32 ± 11	35 ± 27	29 ± 12	0.025
Red and processed meat (g/d)	41 ± 28	36 ± 20	0.01	45 ± 26	40 ± 27	34 ± 17	0.003
Fish (g/d)	9.3 ± 12	9.1 ± 9	0.01	10 ± 11	10 ± 11	8 ± 9	0.427
Egg (g/d)	26 ± 17	27 ± 20	0.55	24 ± 13	27 ± 22	26 ± 16	0.631
Legumes and nuts (g/d)	40 ± 23	46 ± 20	0.008	38 ± 18	39 ± 19	50 ± 22	< 0.001
Dairy (g/d)	309 ± 117	355 ± 131	< 0.001	312 ± 129	327 ± 122	356 ± 133	0.041
Refined grains (g/d)	501 ± 175	421 ± 182	< 0.001	550 ± 237	461 ± 168	416 ± 176	< 0.001
Whole grains (g/d)	177 ± 134	150 ± 108	0.04	114 ± 86	144 ± 120	181 ± 118	0.001
Salt (g/d)	5.8 ± 2	6.3 ± 2	0.01	6.3 ± 2	6.5 ± 2	5.8 ± 2	0.003
Sugar sweetened beverages (g/d)	79 ± 67	83 ± 74	0.54	116 ± 99	95 ± 77	63 ± 54	< 0.001

*Obtained by ANOVA

Multivariable-adjusted ORs for glioma across categories of the HLS are shown in Table 3. After adjustment for age and sex, we found that individuals with the highest HLS score were 55% less likely to have glioma compared with those with the lowest score (OR: 0.45; 95% CI: 0.22, 0.92). Additional controlling for other potential confounders made the association stronger (OR: 0.28; 95%CI: 0.12, 0.66). After further adjustment for cooking methods, we found that adherence to HLS score was protectively associated with reduced chance of glioma (OR: 0.29; 95%CI: 0.12, 0.70).

**Table 3:** Multivariable-adjusted ratios for glioma across different categories of the Healthy lifestyle (HLS) score

	Healthy lifestyle score			
	0 (n = 38)	1 (n = 161)	2 (n = 184)	
	OR	OR 95% CI	OR 95% CI	P trend
Crude	1.00	0.59 0.29–1.21	0.45 0.22–0.92	0.028
Model I	1.00	0.60 0.29–1.22	0.45 0.22–0.92	0.029
Model II	1.00	0.59 0.26–1.34	0.28 0.12–0.66	0.001
Model III	1.00	0.63 0.26–1.50	0.29 0.12–0.70	0.001

Model I: Adjusted for age and sex

Model II: Further controlled for marital status (married/single/divorced), education (university graduated/ non-university education), high-risk occupation (farmer/ non-farmer), high-risk residential area (yes/no), duration of cell phone use (continues), supplement use (yes/no), drug use (yes/no), smoking status (smoker/non-smoker), exposure to chemicals (yes/no), personal hair dye use (yes/no), family history of cancer (yes/no), family history of glioma (yes/no), history of exposure to the radiographic X-ray (yes/no), history of head trauma (yes/no), history of allergy (yes/no), history of hypertension (yes/no)

Model III: Additionally adjusted for frequent fried food intake (yes/no), frequent use of barbecue (yes/no), canned foods (yes/no), and microwave (yes/no)

Multivariate-adjusted odds ratio for glioma across different levels of individual components of healthy lifestyle score revealed that there was no significant association between adhering to a healthy diet and odds of glioma (OR: 0.67; 95%CI: 0.43, 1.04) in crude model. After controlling for potential confounders, individuals with the healthy diet had 54% lower odds of glioma than those with a non-healthy diet (OR: 0.46; 95%CI: 0.26, 0.80). No significant associations were seen between physical activity levels or BMI status and glioma in crude (For physical activity: OR: 0.98; 95% CI: 0.62, 1.54 and for BMI status: OR: 0.74; 95% CI: 0.41, 1.31) or multivariable-adjusted model (For physical activity: OR: 0.84; 95%CI: 0.54, 1.29 and for BMI status: OR: 0.76; 95% CI: 0.43, 1.33).

## Discussion

In this case-control study, we found a significant inverse association between healthy lifestyle score and risk of glioma among Iranian population, even after adjustment for potential confounders. In terms of individual components of healthy lifestyle, we found a significant inverse association between a healthy diet and chance of glioma. To the best of our knowledge, this study is the first observational study examining the association between combined healthy lifestyle score and risk of glioma.

Several risk factors for glioma were reported in prospective studies [16, 47]. In a study by Kuan et al., it has shown that higher risk of glioma was associated with increased intakes of total fruit, citrus fruit, and fiber and healthy dietary patterns, but these relations were generally null after excluding the first 5 years of follow-up. However, in this report, there were some evidences of heterogeneity of results by study or sex [47]. Despite the role of genetic factors in the initiation and progression of glioma, it seems that several modifiable environmental factors are also involved in its incidence [11]. For instance, adherence to an anti-inflammatory or pro-inflammatory diet might influence the risk of glioma [9]. Saunders et al., have identified some potential modifiable factors and their associations with glioma through Mendelian randomization (MR) analysis. They found wide ranges of causal network for glioma including diet related variables. BMI, fat mass, life style, some dietary micro-nutrients were associated with the risk of glioma [48]. It should be mentioned that MR analysis can reduce many of the limitations of observational studies; therefore, it can provide more exact associations. We found that combined healthy lifestyle factors might reduce the odds of glioma. Although so far no study has linked combined healthy lifestyle score to the risk of glioma, several investigations have examined this score with other cancers and chronic diseases. Kenfield et al. indicated that adherence to a healthy lifestyle reduced the risk of lethal prostate cancer [20]. Jiao et al. showed in prospective study that combining 5 modifiable lifestyle factors including Mediterranean dietary pattern, normal BMI, regular physical activity, no-smoking and limited alcohol use could substantially reduce the risk of developing pancreatic cancer [21]. Low estrogen-related lifestyle score that was constructed based on low alcohol consumption, low body weight, and high levels of physical activity was associated with a reduced risk of postmenopausal breast cancer [19]. Given the high mortality rate of this condition, even the minimal protective effects would greatly influence the burden of disease in general population. Although no study is available examining the association of the whole lifestyle factors with glioma, individual components of lifestyle, including diet, smoking and physical activity have earlier been assessed in relation to glioma [10–14]. Considering individual components of lifestyle score, we found an inverse association between healthy diet and odds of glioma. Adherence to DASH diet was inversely associated with glioma risk [38]. The protective association of healthy dietary patterns with glioma might be explained by the specific nutrient content of these diets including high amounts of anti-oxidants. Given the high content of vitamin E, vitamin A, beta-carotene, calcium, molybdenum, mono-saturated fatty acid and poly-unsaturated fatty acids in these diets, such a diet can inversely influence the risk of glioma [37]. In terms of physical activity, a meta-analysis on observational studies revealed a weak protective association between high levels of physical activity and risk of glioma [49]. Despite some significant associations with glioma in some case-control studies [50], findings from prospective studies, in line with ours, did not reveal an association between physical activity and risk of glioma [51]. Physical activity might influence the process of carcinogenesis through lowering free insulin and IGF-1 levels; however, it seems that the binding of these molecules to their proteins is very low in glioma [51]. Adiposity and increased BMI as one of HLS was extensively studied in relation to glioma. In a most recent meta-analysis, null association was seen between BMI and risk of glioma [49]. This was also seen in most prospective studies [51]. It seems that early life BMI during childhood is much more important than adulthood BMI in determining the risk of glioma. It is shown that weight and energy balance during early life might alter adult risk of brain tumors [51]. As the nature of this study was case-control, it was impossible for us to determine the contribution of childhood BMI to the risk. We did not include smoking in our HLS construction as previous meta-analysis on cohort studies did not introduce smoking as a strong risk factor for glioma. However, we adjusted for smoking in the statistical analysis.

Given the role of inflammatory pathways and oxidative stress in carcinogenesis and glioma, one might assume that healthy lifestyle factors including healthy diet, high physical activity and low BMI may influence the risk of glioma through modulation of inflammatory and oxidative stress pathways. In addition, these components mostly suppress insulin-like growth factor signaling which acts in glioma initiation and promotion [51].

Being the first report on combined healthy lifestyle factors and risk of glioma and considering several confounding factors into account are strengths of this study. However, there are some limitations that should be considered in interpretation of results. Due to the case-control design of this study, causality cannot be inferred. Recall bias and memory loss are other limitations of the study. We are aware that the intakes of people can be affected by their current disease and confound their reports. Due to these limitations, we recruited only the new cases of the glioma patients in this study. Moreover, we asked about their food intakes and cooking methods from their wives, parents, relatives or care takers that prepare their foods as needed. Also, we had no relevant data on dietary intakes of trans fat and did not consider it in the construction of score or in the models. Although the aim of this study was the prevention and people who have a healthy diet, probably have less likely odds of glioma, due to case-control design causality should be interpreted cautiously. The dietary intakes were measured by FFQ just a year before the diagnosis of glioma. However, most of the cancers develop long before. Therefore, the measurement of dietary intakes just 1 year prior to diagnosis is questionable. Some glioma patients might suffer from memory dysfunction. This can also lead to some sort of measurement error. However, we asked persons involved in food preparation to help reporting the dietary intake. It must be kept in mind that we did not include smoking in our HLS because findings from earlier studies did not introduce smoking as a risk factor for glioma [52–55]. Although glioma cases were diagnosed by conventional imaging techniques including magnetic resonance imaging (MRI) and computed tomography (CT) scan, it might be better to use of the combination of positron emission tomography (PET)/MRI scan for primary diagnosis and evaluation of patients.

## Conclusion

Life style related factors have been contributed with the risk of glioma. However, there were limited data on the relation of healthy life style score and chance of glioma. This is in particular relevant to Middle Eastern region where lifestyle variables are different from the other parts of the world. Understanding this association can move forward the science in terms of prevention of glioma. In conclusion, we found evidence indicating that adherence to healthy lifestyle was associated with a lower odds of glioma. However, future prospective investigations are needed to confirm the causality.

## Data Availability

Not applicable.

## References

[1] Ostrom QT, Gittleman H, Liao P, Vecchione-Koval T, Wolinsky Y, Kruchko C (2017). CBTRUS Statistical Report: Primary brain and other central nervous system tumors diagnosed in the United States in 2010–2014. Neuro Oncol.

[2] Behzadnia H, Alijani B, Emamhadi M, Yousefzadeh-Chabok S, Haghdoost Z (2015). Glioblastoma Multiforme: A Single Hospital Experience. IrJNS.

[3] Jazayeri SB, Rahimi-Movaghar V, Shokraneh F, Saadat S, Ramezani R (2013). Epidemiology of primary CNS tumors in Iran: a systematic review. Asian Pac J Cancer Prev.

[4] Florian IS, Ungureanu G, Berce C (2013). Risk factors for gliomas. An extensive review Romanian Neurosurgery.

[5] Schlehofer B, Hettinger I, Ryan P, Blettner M, Preston-Martin S, Little J (2005). Occupational risk factors for low grade and high grade glioma: results from an international case control study of adult brain tumours. Int J Cancer.

[6] Hu J, Johnson KC, Mao Y, Guo L, Zhao X, Jia X (1998). Risk factors for glioma in adults: a case-control study in Northeast China. Cancer Detect Prev.

[7] Steck PA, Hadi A, Lotan R, Yung WK (1990). Inhibition of epidermal growth factor receptor activity by retinoic acid in glioma cells. J Cell Biochem.

[8] De Vleeschouwer S (2017). Glioblastoma.

[9] Kyritsis AP, Bondy ML, Levin VA (2011). Modulation of glioma risk and progression by dietary nutrients and anti-inflammatory agents. Nutr Cancer.

[10] Roomi MW, Niedzwiecki A, Rath M (2018). Scientific evaluation of dietary factors in Cancer. J Nutri Med Diet Care.

[11] Bielecka J, Markiewicz-˙Zukowska R. (2020). The influence of nutritional and lifestyle factors on glioma incidence. Nutrients.

[12] Little RB, Madden MH, Thompson RC, Olson JJ, Larocca RV, Pan E (2013). Anthropometric factors in relation to risk of glioma. Cancer Causes Control.

[13] Benson VS, Pirie K, Green J, Casabonne D, Beral V (2008). Lifestyle factors and primary glioma and meningioma tumours in the million women study cohort. Br J Cancer.

[14] Nelson JS, Burchfiel CM, Fekedulegn D, Andrew ME (2012). Potential risk factors for incident glioblastoma multiforme: the Honolulu heart program and Honolulu-Asia aging study. J Neuro-Oncol.

[15] Scheurer ME, El-Zein R, Thompson PA, Aldape KD, Levin VA, Gilbert MR (2008). Long-term anti-inflammatory and antihistamine medication use and adult glioma risk. Cancer Epidemiol Biomark Prev.

[16] Wrensch M, Minn Y, Chew T, Bondy M, Berger MS (2002). Epidemiology of primary brain tumors: current concepts and review of the literature. Neuro-Oncology.

[17] Li H, Peng X, Zong Q, Zhang K, Wang M, Liu Y (2016). Cigarette smoking and risk of adult glioma: a meta-analysis of 24 observational studies involving more than 2.3 million individuals. Onco Targets Ther.

[18] Yun JE, Won S, Kimm H, Jee SH (2012). Effects of a combined lifestyle score on 10-year mortality in Korean men and women: a prospective cohort study. BMC Public Health.

[19] Guinter MA, McLain AC, Merchant AT, Sandler DP, Steck SE (2018). An estrogen-related lifestyle score is associated with risk of postmenopausal breast cancer in the PLCO cohort. Breast Cancer Res Treat.

[20] Kenfield SA, Batista JL, Jahn JL, Downer MK, Van Blarigan EL, Sesso HD, et al. Development and application of a lifestyle score for prevention of lethal prostate Cancer. J Natl Cancer Inst. 2015;17:108(3). 10.1093/jnci/djv329 PMC5964905 26577654

[21] Jiao L, Mitrou PN, Reedy J, Graubard BI, Hollenbeck AR (2009). A combined healthy lifestyle score and risk of pancreatic Cancer - a large cohort study. Arch Intern Med.

[22] Díaz-Gutiérrez J, Ruiz-Canela M, Gea A, Fernández-Montero A, Martínez-González MÁ. Association between a healthy lifestyle score and the risk of cardiovascular disease in the SUN cohort. Rev Esp Cardiol (Engl Ed). 2017;26. 10.1016/j.rec.2017.10.038 29287797

[23] Sibai AM, Nasreddine L, Mokdad AH, Adra N, Tabet M, Hwalla N (2010). Nutrition transition and cardiovascular disease risk factors in Middle East and North Africa countries: reviewing the evidence. Ann Nutr Metab.

[24] Naja F, Nasreddine L, Awada S, Ahmad RES, Hwalla N (2019). Nutrition in the prevention of breast Cancer: a middle eastern perspective. Front Public Health.

[25] Al Jawaldeh A, Al-Jawaldeh H (2018). Fat intake reduction strategies among children and adults to eliminate obesity and non-communicable diseases in the eastern Mediterranean region. Children..

[26] Bahreynian M, Esmaillzadeh A (2012). Quantity and quality of carbohydrate intake in Iran: a target for nutritional intervention. Archives of Iranian Medicine.

[27] Ng M, Freeman MK, Fleming TD, Robinson M, Dwyer-Lindgren L, Thomson B (2014). Smoking prevalence and cigarette consumption in 187 countries, 1980-2012. JAMA.

[28] Hagen EH, Garfield MJ, Sullivan RJ (2016). The low prevalence of female smoking in the developing world: gender inequality or maternal adaptations for fetal protection?. Evol Med Public Health.

[29] Tobacco-WHO| www.who.int.

[30] Eloul L, Ambusaidi A, Al-Adawi S (2009). Silent epidemic of depression in women in the Middle East and North Africa region: emerging tribulation or fallacy?. Sultan Qaboos Univ Med J.

[31] Sayar K, Kose S (2012). Psychopathology and depression in the Middle East. Journal of Mood Disorders.

[32] Razzak HA, Harbi A, Ahli S (2019). Depression: prevalence and associated risk factors in the United Arab Emirates. Oman Med J.

[33] Chaabane S, Chaabna K, Abraham A, Mamtani R, Cheema S (2020). Physical activity and sedentary behaviour in the Middle East and North Africa: an overview of systematic reviews and meta-analysis. Sci Rep.

[34] Donnelly TT, Al-Thani Al M, Benjamin K, Al-Khater Al H, Fung TS, Ahmedna M, Welch A (2018). Arab female and male perceptions of factors facilitating and inhibiting their physical activity: findings from a qualitative study in the Middle East. PLoS One.

[35] Shuval K, Weissblueth E, Amira A, Brezis M, Faridi Z, Ali A (2008). The role of culture, environment, and religion in the promotion of physical activity among Arab Israelis. Prev Chronic Dis.

[36] Ebrahimpour-Koujan S, Shayanfar M, Benisi-Kohansal S, Mohammad-Shirazi M, Sharifi G, Esmaillzadeh A (2019). Adherence to low carbohydrate diet in relation to glioma: a case-control study. Clin Nutr.

[37] Malmir H, Shayanfar M, Mohammad-Shirazi M, Tabibi H, Sharifi G, Esmaillzadeh A (2019). Patterns of nutrients intakes in relation to glioma: a case-control study. Clin Nutr.

[38] Benisi-Kohansal S, Shayanfar M, Mohammad-Shirazi M, Tabibi H, Sharifi G, Saneei P, Esmaillzadeh A (2016). Adherence to the dietary approaches to stop hypertension-style diet in relation to glioma: a case-control study. Br J Nutr.

[39] Malekshah AF, Kimiagar M, Saadatian-Elahi M, Pourshams A, Nouraie M, Goglani G (2006). Validity and reliability of a new food frequency questionnaire compared to 24 h recalls and biochemical measurements: pilot phase of Golestan cohort study of esophageal cancer. Eur J Clin Nutr.

[40] Chiuve SE, Fung TT, Rimm EB, Hu FB, McCullough ML, Wang M (2012). Alternative dietary indices both strongly predict risk of chronic disease. J Nutr.

[41] Willett WC (1998). Nutritional epidemiology.

[42] Fritz A, Percy C, Jack A, et al. International classification of diseases for oncology (ICD-O). Third Edition World Health Organization (WHO). 2012.

[43] Ruder AM, Carreón T, Butler MA, Calvert GM, Davis-King KE, Waters MA (2009). Exposure to farm crops, livestock, and farm tasks and risk of glioma: the upper Midwest health study. Am J Epidemiol.

[44] Morgan LL, Miller AB, Sasco A, Davis DL (2015). Mobile phone radiation causes brain tumors and should be classified as a probable human carcinogen (2A) (review). Int J Oncol.

[45] Kheifets LI (2001). Electric and magnetic field exposure and brain cancer: a review. Bioelectromagnetics.

[46] Zhi WJ, Wang LF, Hu XJ (2017). Recent advances in the effects of microwave radiation on brains. Mil Med Res.

[47] Kuan AS, Green J, Kitahara CM, De González AB, Key T, Reeves GK (2019). Diet and risk of glioma: combined analysis of 3 large prospective studies in the UK and USA. Neuro-Oncology.

[48] Saunders CN, Cornish AJ, Kinnersley B, Law PJ, Claus EB, Il'yasova D (2020). Lack of association between modifiable exposures and glioma risk: a Mendelian randomization analysis. Neuro-Oncology.

[49] Niedermaier T, Behrens G, Schmid D, Schlecht I, Fischer B, Leitzmann MF (2015). Body mass index, physical activity, and risk of adult meningioma and glioma: a meta-analysis. Neurology..

[50] Cabaniols C, Giorgi R, Chinot O, Ferahta N, Spinelli V, Alla P (2011). Links between private habits, psychological stress and brain cancer: a case-control pilot study in France. J Neuro-Oncol.

[51] Michaud DS, Bové G, Gallo V, Schlehofer B, Tjønneland A, Olsen A (2011). Anthropometric measures, physical activity, and risk of glioma and meningioma in a large prospective cohort study. Cancer Prev Res (Phila).

[52] Guo X, Wang Y (2016). Does smoking increase the risk of developing glioma? A meta-analysis based on case-control studies. J Cancer Res Ther.

[53] Shao C, Zhao W, Qi Z, He J. Smoking and glioma risk: evidence from a Meta-analysis of 25 observational studies. Medicine (Baltimore) 2016; 95(2): e2447. 10.1097/MD.0000000000002447 PMC4718259 26765433

[54] Ahn S, Han KD, Park YM, Bae JM, Kim SU, Jeun SS (2020). Cigarette smoking is associated with increased risk of malignant gliomas: a Nationwide population-based cohort study. Cancers.

[55] Braganza MZ, Rajaraman P, Park Y, Inskip PD, Freedman ND, Hollenbeck AR (2014). Cigarette smoking, alcohol intake, and risk of glioma in the NIH-AARP diet and health study. Br J Cancer.

